# Neuroendoscopical management of intracranial lesions: application of endovascular stent for cystoventriculostomy (New Technique)

**Published:** 2012-11

**Authors:** Mohammad Ghorbani, Enayatollah Abbasnejad, Mahmoud Ramak Hashemi, Reza Mollahosseini, Maziar Azar

**Affiliations:** ^*a*^Department of Neurosurgery, Firoozgar Hospital, Tehran University of Medical Sciences, Tehran, Iran.

## Abstract

**Background:**

Neuroendoscopic system is the best choice for detection and assessment of intracranial cysts and intraventricular tumors and cysts. There are some reasons and features for neuroendoscopy to be the number-one option for these dysfunctions such as entrapment of cerebrospinal fluid (CSF) inside the arachnoid cysts, location of intraventricular tumors within the CSF, and generally any ventricular enlargement because of CSF pathway obstruction. These features provide optimal space for visualization and working by neuroendoscopy. However, in the context of recent technologies such as robotic assisted neuroendoscopy and frameless stereotactic based neuroendoscopy, the ventricular enlargement is no longer an essential prerequisite for neuroendoscopy practice.

This study presents an overview of history and current advancement of neuroendoscopy as a safe and effective management modality for the treatment of a variety of intracranial disorders, its indications and limitations which underpin fundamental basis of modern neurosurgery. Furthermore, we present our new technique for the treatment of intracranial arachnoid cyst using endovascular stent for patency maintenance of opening pathway.

**Keywords:**

Neuroendoscopy, Arachnoid cyst, Endoscope, Intraventricular lesions, Endovascular stent


					Volume 4, Suppl. 1 Nov 2012
Publisher: Kermanshah University of Medical Sciences
URL: http://www.jivresearch.org
Copyright:
Congress of Iranian Neurosurgeons, 14-16 November 2012, Kermanshah, Iran
Paper No. 30
Neuroendoscopical management of intracranial lesions:
application of endovascular stent for cystoventriculostomy
(New Technique)
Mohammad Ghorbani a ,*
, Enayatollah Abbasnejad a
, Mahmoud Ramak Hashemi a
, Reza Mollahosseini a
,
Maziar Azar a
a
Department of Neurosurgery, Firoozgar Hospital, Tehran University of Medical Sciences, Tehran, Iran.
Abstract:
Background: Neuroendoscopic system is the best choice for detection and assessment of intracranial cysts and
intraventricular tumors and cysts. There are some reasons and features for neuroendoscopy to be the number-one
option for these dysfunctions such as entrapment of cerebrospinal fluid (CSF) inside the arachnoid cysts, location of
intraventricular tumors within the CSF, and generally any ventricular enlargement because of CSF pathway
obstruction. These features provide optimal space for visualization and working by neuroendoscopy. However, in the
context of recent technologies such as robotic assisted neuroendoscopy and frameless stereotactic based
neuroendoscopy, the ventricular enlargement is no longer an essential prerequisite for neuroendoscopy practice.
This study presents an overview of history and current advancement of neuroendoscopy as a safe and effective
management modality for the treatment of a variety of intracranial disorders, its indications and limitations which
underpin fundamental basis of modern neurosurgery. Furthermore, we present our new technique for the treatment of
intracranial arachnoid cyst using endovascular stent for patency maintenance of opening pathway.
Keywords:
Neuroendoscopy, Arachnoid cyst, Endoscope, Intraventricular lesions, Endovascular stent,
* Corresponding Author at:
Mohammad Ghorbani: Department of Neurosurgery, Firoozgar Hospital, Tehran University of Medical Sciences, Tehran, Iran. Tel: 09126782570
Email: mohghorbani@gmail.com, (Ghorbani M.).

				

